# 3,5-Bis(4-chloro­benzyl­idene)-1-methyl­piperidin-4-one

**DOI:** 10.1107/S1600536811006994

**Published:** 2011-03-02

**Authors:** Volodymyr V. Nesterov, Sergey S. Sarkisov, Vladimir Shulaev, Vladimir N. Nesterov

**Affiliations:** a Department of Natural Sciences, New Mexico Highlands University, Las Vegas, NM 87701, USA; b SSS Optical Technologies, LLC, 515 Sparkman Drive, Suite 122, Huntsville, AL 35816, USA; c Department of Biological Sciences, University of North Texas, Denton, TX 76203, USA; d Department of Chemistry, University of North Texas, Denton, TX 76203, USA

## Abstract

In the title mol­ecule, C_20_H_17_Cl_2_NO, the central heterocyclic ring adopts a flattened boat conformation. The dihedral angles between the planar part of this central heterocyclic ring [maximum deviation = 0.004 (1) Å] and the two almost planar side-chain fragments [maximum deviations = 0.015 (1) and 0.019 (1) Å], that include the aromatic ring and bridging atoms, are 18.1 (1) and 18.0 (1)°. In the crystal, pairs of weak inter­molecular C—H⋯O hydrogen bonds link mol­ecules into inversion dimers that form stacks along the *a* axis. The structure is further stabilized by weak inter­molecular C—H⋯π inter­actions involving the benzene rings.

## Related literature

For non-linear optical organic compounds with two-photon absorption properties and potential biophotonic materials, see: Nesterov *et al.* (2003 ▶); Nesterov (2004 ▶); Sarkisov *et al.* (2005 ▶). For the biological importance of 4-piperidone, see: Jia *et al.* (1988 ▶, 1989 ▶); Dimmock *et al.* (2001 ▶). For the synthesis of the title compound, see: Dimmock *et al.* (2001 ▶). For related structures, see: Nesterov (2004 ▶); Nesterov *et al.* (2003 ▶, 2007*a* ▶,*b* ▶,*c* ▶, 2008 ▶). For weak hydrogen bonds, see: Desiraju & Steiner (1999 ▶). For the van der Waals radius of the H atom, see: Rowland & Taylor (1996 ▶).
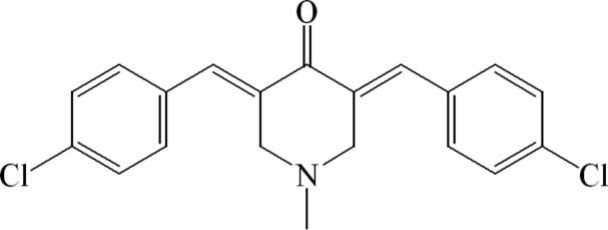


## Experimental

### 

#### Crystal data

C_20_H_17_Cl_2_NO
*M* *_r_* = 358.25
Monoclinic, 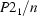

*a* = 5.4568 (11) Å
*b* = 13.916 (3) Å
*c* = 22.289 (4) Å
β = 90.847 (3)°
*V* = 1692.4 (6) Å^3^
*Z* = 4
Mo *K*α radiation
μ = 0.39 mm^−1^
*T* = 100 K
0.23 × 0.18 × 0.08 mm

#### Data collection

Bruker SMART APEX II CCD diffractometer
Absorption correction: multi-scan (*SADABS*; Bruker, 2001 ▶) *T*_min_ = 0.916, *T*_max_ = 0.970
14890 measured reflections
3461 independent reflections
2830 reflections with *I* > 2σ(*I*)
*R*_int_ = 0.042

#### Refinement

*R*[*F* ^2^ > 2σ(*F* ^2^)] = 0.034
*wR*(*F* ^2^) = 0.080
*S* = 1.03
3461 reflections
218 parameters
H-atom parameters constrained
Δρ_max_ = 0.27 e Å^−3^
Δρ_min_ = −0.28 e Å^−3^

### 

Data collection: *APEX2* (Bruker, 2007 ▶); cell refinement: *SAINT* (Bruker, 2007 ▶); data reduction: *SAINT*; program(s) used to solve structure: *SHELXS97* (Sheldrick, 2008 ▶); program(s) used to refine structure: *SHELXL97* (Sheldrick, 2008 ▶); molecular graphics: *SHELXTL* (Sheldrick, 2008 ▶); software used to prepare material for publication: *SHELXTL*.

## Supplementary Material

Crystal structure: contains datablocks I, global. DOI: 10.1107/S1600536811006994/su2255sup1.cif

Structure factors: contains datablocks I. DOI: 10.1107/S1600536811006994/su2255Isup2.hkl

Additional supplementary materials:  crystallographic information; 3D view; checkCIF report

## Figures and Tables

**Table 1:** Hydrogen-bond geometry (Å, °) *Cg*1 and *Cg*2 are the centroids of the C15–C20 and C8–C13 rings, respectively.

*D*—H⋯*A*	*D*—H	H⋯*A*	*D*⋯*A*	*D*—H⋯*A*
C9—H9*A*⋯O1^i^	0.95	2.47	3.210 (2)	135
C12—H12*A*⋯*Cg*1^ii^	0.95	2.72	3.439 (2)	133
C19—H19*A*⋯*Cg*2^iii^	0.95	2.73	3.432 (2)	131

Symmetry codes: (i) 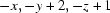; (ii) 
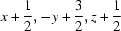
; (iii) 
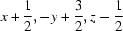
.

**Fig. 1.**
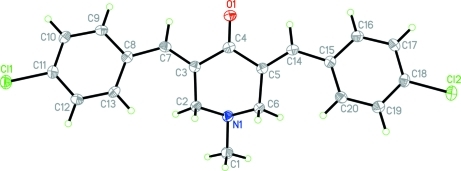
View of the molecular structure of the title molecule, with thermal ellipsoids drawn at the 50% probability level.

**Fig. 2.**
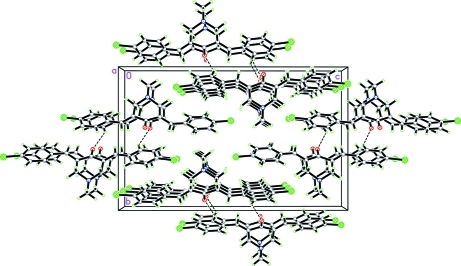
Projection of the crystal packing of the title compound along the a-axis. Dashed lines denote weak intermolecular C—H···O hydrogen bonds.

## supplementary crystallographic information

### Comment

Continuing our work on the synthesis and structural investigations of nonlinear optical organic compounds with two-photon absorption properties and potential biophotonic materials (Nesterov *et al.*, 2003; Nesterov, 2004; Nesterov *et al.*, 2007*a*-c; Nesterov *et al.*, 2008; Sarkisov *et al.*, 2005), we investigated the crystal structure of the title compound. This compound belongs to a group that has shown anticancer activity (Jia *et al.*, 1988; Jia *et al.*, 1989; Dimmock *et al.*, 2001). It may also find application as an agent for locating cancer cells with two photon excited fluorescence and as potential agent for a photodynamic treatment of cancer (Nesterov *et al.*, 2003; Sarkisov *et al.*, 2005).

The molecular structure of the title molecule is illustrated in Fig. 1. The central heterocycle adopts a flattened boat conformation: atoms N1 and C4 lie -0.723 (1) and -0.205 (1) Å, respectively, out of the central C_4_ plane [planar within 0.004 (1) Å]. Dihedral angles between the flat part of the heterocycle (atoms C2,C3,C5,C6) and the two almost planar fragments that include the Ph-ring and the bridging atoms are 18.1 (1) and 18.0 (1)° for (C7-C13) and (C14-C20), respectively. Such nonplanarity might partly be caused by the presence of short intramolecular contacts H2B···H13A and H6A···H20A with distances 2.16 and 2.15 Å, that are somewhat shorter than the doubled van der Waals radii of the H atom (Rowland & Taylor, 1996). Atom N1 in the piperidone ring has a pyramidal coordination with the sum of bond angles equal to 329.8 (1)°, while the methyl substituent connected to it occupies an equatorial position.

In the crystal there are weak intermolecular C—H···O (H9A···O1 2.47 Å) contacts (Table 1) that could be considered as weak hydrogen bonds (Desiraju & Steiner, 1999). Such H-bonds link the molecules into dimers, centered about an inversion center, that form stacks along the *a*-axis (Fig. 2). The structure of the molecule is further stabilized by weak intermolecular C-H···π-interactions involving the benzene rings (Table 1).

### Experimental

The title compound was obtained according to the literature procedure (Dimmock *et al.*, 2001) by the reaction of *p*-chlorobenzaldehyde with 1-methyl-4-piperidone. The precipitate obtained was isolated and recrystallized from ethanol/acetonitrile [v/v = 50/50]; Mp. 442 K, yield 87%). The title compound was characterized by ^1^H and ^13^C NMR spectroscopy.

### Refinement

All C-bound H atoms were placed in idealized positions and allowed to ride on their parent atom: C—H = 0.95, 0.98 and 0.99 Å for CH, CH_3_ and CH_2_ H-atoms, respectively, with *U*_iso_(H) = k × *U*_eq_(C), where k = 1.5 for CH_3_ H-atoms, and k = 1.2 for all other H-atoms.

### Crystal data

**Table:**

C_20_H_17_Cl_2_NO	*F*(000) = 744
*M**_r_* = 358.25	*D*_x_ = 1.406 Mg m^−^^3^
Monoclinic, *P*2_1_/*n*	Mo *K*α radiation, λ = 0.71073 Å
Hall symbol: -P 2yn	Cell parameters from 2327 reflections
*a* = 5.4568 (11) Å	θ = 2.4–25.2°
*b* = 13.916 (3) Å	µ = 0.39 mm^−^^1^
*c* = 22.289 (4) Å	*T* = 100 K
β = 90.847 (3)°	Plate, yellow
*V* = 1692.4 (6) Å^3^	0.23 × 0.18 × 0.08 mm
*Z* = 4	

### Data collection

**Table:**

Bruker SMART APEX II CCD diffractometer	3461 independent reflections
Radiation source: fine-focus sealed tube	2830 reflections with *I* > 2σ(*I*)
graphite	*R*_int_ = 0.042
ω scans	θ_max_ = 26.4°, θ_min_ = 1.8°
Absorption correction: multi-scan (*SADABS*; Bruker, 2001)	*h* = −6→6
*T*_min_ = 0.916, *T*_max_ = 0.970	*k* = −17→17
14890 measured reflections	*l* = −27→27

### Refinement

**Table:**

Refinement on *F*^2^	Primary atom site location: structure-invariant direct methods
Least-squares matrix: full	Secondary atom site location: difference Fourier map
*R*[*F*^2^ > 2σ(*F*^2^)] = 0.034	Hydrogen site location: inferred from neighbouring sites
*wR*(*F*^2^) = 0.080	H-atom parameters constrained
*S* = 1.03	*w* = 1/[σ^2^(*F*_o_^2^) + (0.030*P*)^2^ + 0.850*P*] where *P* = (*F*_o_^2^ + 2*F*_c_^2^)/3
3461 reflections	(Δ/σ)_max_ = 0.001
218 parameters	Δρ_max_ = 0.27 e Å^−^^3^
0 restraints	Δρ_min_ = −0.28 e Å^−^^3^

### Special details

**Table:**

Geometry. All e.s.d.'s (except the e.s.d. in the dihedral angle between two l.s. planes) are estimated using the full covariance matrix. The cell e.s.d.'s are taken into account individually in the estimation of e.s.d.'s in distances, angles and torsion angles; correlations between e.s.d.'s in cell parameters are only used when they are defined by crystal symmetry. An approximate (isotropic) treatment of cell e.s.d.'s is used for estimating e.s.d.'s involving l.s. planes.
Refinement. Refinement of *F*^2^ against ALL reflections. The weighted *R*-factor *wR* and goodness of fit *S* are based on *F*^2^, conventional *R*-factors *R* are based on *F*, with *F* set to zero for negative *F*^2^. The threshold expression of *F*^2^ > σ(*F*^2^) is used only for calculating *R*-factors(gt) *etc*. and is not relevant to the choice of reflections for refinement. *R*-factors based on *F*^2^ are statistically about twice as large as those based on *F*, and *R*- factors based on ALL data will be even larger.

### Fractional atomic coordinates and isotropic or equivalent isotropic displacement parameters (Å^2^)

**Table:**

	*x*	*y*	*z*	*U*_iso_*/*U*_eq_	
Cl1	0.64148 (8)	0.85150 (3)	0.742372 (19)	0.02375 (12)	
Cl2	0.64623 (9)	0.87411 (3)	0.01133 (2)	0.02727 (13)	
O1	0.1157 (2)	0.92396 (9)	0.37523 (5)	0.0245 (3)	
N1	0.5768 (3)	0.71047 (10)	0.37453 (6)	0.0191 (3)	
C1	0.7423 (4)	0.62787 (13)	0.37437 (8)	0.0245 (4)	
H1A	0.7070	0.5863	0.4087	0.037*	
H1B	0.9123	0.6503	0.3773	0.037*	
H1C	0.7187	0.5916	0.3370	0.037*	
C2	0.6145 (3)	0.76651 (12)	0.42936 (8)	0.0187 (4)	
H2A	0.7803	0.7953	0.4295	0.022*	
H2B	0.6023	0.7239	0.4648	0.022*	
C3	0.4243 (3)	0.84484 (12)	0.43294 (8)	0.0172 (4)	
C4	0.3040 (3)	0.87585 (12)	0.37573 (8)	0.0188 (4)	
C5	0.4283 (3)	0.84936 (12)	0.31884 (8)	0.0170 (4)	
C6	0.6202 (3)	0.77144 (12)	0.32221 (8)	0.0183 (4)	
H6A	0.6132	0.7321	0.2852	0.022*	
H6B	0.7852	0.8007	0.3255	0.022*	
C7	0.3539 (3)	0.89000 (12)	0.48316 (8)	0.0181 (4)	
H7A	0.2281	0.9364	0.4772	0.022*	
C8	0.4384 (3)	0.87971 (12)	0.54539 (8)	0.0176 (4)	
C9	0.2897 (3)	0.91907 (12)	0.59029 (8)	0.0190 (4)	
H9A	0.1438	0.9519	0.5788	0.023*	
C10	0.3496 (3)	0.91133 (13)	0.65039 (8)	0.0201 (4)	
H10A	0.2454	0.9374	0.6800	0.024*	
C11	0.5648 (3)	0.86468 (12)	0.66671 (8)	0.0187 (4)	
C12	0.7215 (3)	0.82827 (12)	0.62392 (8)	0.0194 (4)	
H12A	0.8710	0.7983	0.6358	0.023*	
C13	0.6586 (3)	0.83584 (12)	0.56397 (8)	0.0191 (4)	
H13A	0.7662	0.8109	0.5347	0.023*	
C14	0.3592 (3)	0.89801 (12)	0.26932 (8)	0.0176 (4)	
H14A	0.2328	0.9438	0.2755	0.021*	
C15	0.4450 (3)	0.89257 (12)	0.20763 (8)	0.0174 (4)	
C16	0.2956 (3)	0.93346 (13)	0.16272 (8)	0.0204 (4)	
H16A	0.1495	0.9655	0.1739	0.024*	
C17	0.3543 (3)	0.92871 (13)	0.10259 (8)	0.0206 (4)	
H17A	0.2491	0.9560	0.0728	0.025*	
C18	0.5693 (3)	0.88342 (12)	0.08676 (8)	0.0192 (4)	
C19	0.7270 (3)	0.84500 (12)	0.12966 (8)	0.0199 (4)	
H19A	0.8760	0.8155	0.1181	0.024*	
C20	0.6655 (3)	0.84995 (12)	0.18978 (8)	0.0191 (4)	
H20A	0.7742	0.8241	0.2193	0.023*	

### Atomic displacement parameters (Å^2^)

**Table:**

	*U*^11^	*U*^22^	*U*^33^	*U*^12^	*U*^13^	*U*^23^
Cl1	0.0283 (3)	0.0252 (2)	0.0177 (2)	0.00033 (19)	0.00000 (17)	−0.00001 (17)
Cl2	0.0321 (3)	0.0321 (3)	0.0177 (2)	0.0028 (2)	0.00405 (18)	0.00208 (18)
O1	0.0208 (7)	0.0300 (7)	0.0229 (7)	0.0082 (6)	0.0024 (5)	0.0004 (6)
N1	0.0227 (8)	0.0170 (7)	0.0175 (7)	0.0018 (6)	0.0019 (6)	0.0001 (6)
C1	0.0325 (11)	0.0186 (9)	0.0226 (10)	0.0051 (8)	0.0020 (8)	0.0000 (7)
C2	0.0185 (9)	0.0194 (9)	0.0181 (9)	0.0016 (7)	0.0020 (7)	0.0010 (7)
C3	0.0154 (8)	0.0177 (9)	0.0187 (9)	−0.0027 (7)	0.0025 (7)	0.0016 (7)
C4	0.0177 (9)	0.0164 (9)	0.0224 (9)	−0.0014 (7)	0.0021 (7)	−0.0005 (7)
C5	0.0151 (8)	0.0172 (8)	0.0187 (9)	−0.0016 (7)	−0.0007 (7)	−0.0023 (7)
C6	0.0180 (9)	0.0196 (9)	0.0173 (9)	0.0020 (7)	0.0017 (7)	−0.0005 (7)
C7	0.0161 (8)	0.0173 (8)	0.0209 (9)	0.0005 (7)	0.0016 (7)	0.0022 (7)
C8	0.0190 (9)	0.0154 (8)	0.0186 (9)	−0.0025 (7)	0.0028 (7)	0.0002 (7)
C9	0.0158 (9)	0.0175 (9)	0.0237 (9)	0.0014 (7)	0.0015 (7)	−0.0002 (7)
C10	0.0198 (9)	0.0211 (9)	0.0197 (9)	−0.0013 (7)	0.0049 (7)	−0.0030 (7)
C11	0.0214 (9)	0.0175 (9)	0.0172 (9)	−0.0038 (7)	0.0008 (7)	−0.0005 (7)
C12	0.0161 (9)	0.0191 (9)	0.0229 (9)	0.0004 (7)	−0.0001 (7)	0.0009 (7)
C13	0.0185 (9)	0.0198 (9)	0.0193 (9)	0.0005 (7)	0.0056 (7)	−0.0025 (7)
C14	0.0153 (8)	0.0160 (8)	0.0215 (9)	−0.0001 (7)	0.0001 (7)	−0.0021 (7)
C15	0.0181 (9)	0.0154 (8)	0.0188 (9)	−0.0028 (7)	0.0007 (7)	−0.0010 (7)
C16	0.0189 (9)	0.0195 (9)	0.0229 (9)	0.0014 (7)	0.0011 (7)	0.0008 (7)
C17	0.0204 (9)	0.0213 (9)	0.0199 (9)	−0.0006 (7)	−0.0033 (7)	0.0035 (7)
C18	0.0229 (9)	0.0173 (9)	0.0176 (9)	−0.0046 (7)	0.0027 (7)	0.0009 (7)
C19	0.0175 (9)	0.0192 (9)	0.0230 (9)	−0.0017 (7)	0.0019 (7)	−0.0004 (7)
C20	0.0177 (9)	0.0193 (9)	0.0202 (9)	−0.0005 (7)	−0.0015 (7)	0.0013 (7)

### Geometric parameters (Å, °)

**Table:**

Cl1—C11	1.7412 (18)	C8—C9	1.408 (2)
Cl2—C18	1.7437 (18)	C9—C10	1.378 (2)
O1—C4	1.226 (2)	C9—H9A	0.9500
N1—C2	1.462 (2)	C10—C11	1.386 (2)
N1—C1	1.462 (2)	C10—H10A	0.9500
N1—C6	1.464 (2)	C11—C12	1.386 (2)
C1—H1A	0.9800	C12—C13	1.379 (2)
C1—H1B	0.9800	C12—H12A	0.9500
C1—H1C	0.9800	C13—H13A	0.9500
C2—C3	1.508 (2)	C14—C15	1.461 (2)
C2—H2A	0.9900	C14—H14A	0.9500
C2—H2B	0.9900	C15—C16	1.402 (2)
C3—C7	1.345 (2)	C15—C20	1.404 (2)
C3—C4	1.489 (2)	C16—C17	1.384 (2)
C4—C5	1.493 (2)	C16—H16A	0.9500
C5—C14	1.344 (2)	C17—C18	1.382 (2)
C5—C6	1.509 (2)	C17—H17A	0.9500
C6—H6A	0.9900	C18—C19	1.385 (2)
C6—H6B	0.9900	C19—C20	1.388 (2)
C7—C8	1.462 (2)	C19—H19A	0.9500
C7—H7A	0.9500	C20—H20A	0.9500
C8—C13	1.405 (2)		
			
C2—N1—C1	110.01 (14)	C10—C9—C8	121.96 (16)
C2—N1—C6	109.53 (14)	C10—C9—H9A	119.0
C1—N1—C6	110.27 (13)	C8—C9—H9A	119.0
N1—C1—H1A	109.5	C9—C10—C11	118.66 (16)
N1—C1—H1B	109.5	C9—C10—H10A	120.7
H1A—C1—H1B	109.5	C11—C10—H10A	120.7
N1—C1—H1C	109.5	C10—C11—C12	121.30 (16)
H1A—C1—H1C	109.5	C10—C11—Cl1	119.61 (13)
H1B—C1—H1C	109.5	C12—C11—Cl1	119.09 (14)
N1—C2—C3	109.96 (14)	C13—C12—C11	119.45 (16)
N1—C2—H2A	109.7	C13—C12—H12A	120.3
C3—C2—H2A	109.7	C11—C12—H12A	120.3
N1—C2—H2B	109.7	C12—C13—C8	121.23 (16)
C3—C2—H2B	109.7	C12—C13—H13A	119.4
H2A—C2—H2B	108.2	C8—C13—H13A	119.4
C7—C3—C4	116.71 (16)	C5—C14—C15	131.05 (16)
C7—C3—C2	125.96 (16)	C5—C14—H14A	114.5
C4—C3—C2	117.33 (15)	C15—C14—H14A	114.5
O1—C4—C3	121.64 (16)	C16—C15—C20	117.43 (16)
O1—C4—C5	121.21 (16)	C16—C15—C14	117.37 (15)
C3—C4—C5	117.10 (15)	C20—C15—C14	125.19 (16)
C14—C5—C4	116.58 (15)	C17—C16—C15	122.11 (17)
C14—C5—C6	126.10 (16)	C17—C16—H16A	118.9
C4—C5—C6	117.31 (15)	C15—C16—H16A	118.9
N1—C6—C5	109.62 (14)	C18—C17—C16	118.58 (16)
N1—C6—H6A	109.7	C18—C17—H17A	120.7
C5—C6—H6A	109.7	C16—C17—H17A	120.7
N1—C6—H6B	109.7	C17—C18—C19	121.41 (16)
C5—C6—H6B	109.7	C17—C18—Cl2	119.84 (14)
H6A—C6—H6B	108.2	C19—C18—Cl2	118.75 (14)
C3—C7—C8	130.82 (17)	C18—C19—C20	119.40 (16)
C3—C7—H7A	114.6	C18—C19—H19A	120.3
C8—C7—H7A	114.6	C20—C19—H19A	120.3
C13—C8—C9	117.31 (16)	C19—C20—C15	120.99 (16)
C13—C8—C7	125.35 (16)	C19—C20—H20A	119.5
C9—C8—C7	117.31 (16)	C15—C20—H20A	119.5

### Hydrogen-bond geometry (Å, °)

**Table:**

Cg1 and Cg2 are the centroids of the C15–C20 and C8–C13 rings, respectively.

**Table:**

*D*—H···*A*	*D*—H	H···*A*	*D*···*A*	*D*—H···*A*
C9—H9A···O1^i^	0.95	2.47	3.210 (2)	135
C12—H12A···Cg1^ii^	0.95	2.72	3.439 (2)	133
C19—H19A···Cg2^iii^	0.95	2.73	3.432 (2)	131

Symmetry codes: (i) −*x*, −*y*+2, −*z*+1; (ii) *x*+1/2, −*y*+3/2, *z*+1/2; (iii) *x*+1/2, −*y*+3/2, *z*−1/2.
